# Comparison of Personality Traits, Coping Styles, and Psychiatric Disorders in Adult Suicidal and Non-Suicidal Individuals

**Published:** 2014-07

**Authors:** Farkhondeh Sharif, Azam Parsnia, Arash Mani, Mehrdad Vosoghi, Giti Setoodeh

**Affiliations:** 1 Community Based Psychiatric Care Research Center, Department of Mental Health and Psychiatric Nursing, School of Nursing and Midwifery, Shiraz University of Medical Sciences, Shiraz, Iran;; 2 Department of Mental Health and Psychiatric Nursing, School of Nursing and Midwifery, Shiraz University of Medical Sciences, Shiraz, Iran;; 3 Psychiatry and Behavioral Research Center, Department of Psychiatry, Faculty of Medicine, Shiraz University of Medical Sciences, Shiraz, Iran;; 4 Department of Statistics, Faculty of Medicine, Shiraz University of Medical Sciences, Shiraz, Iran

**Keywords:** Coping styles, Personality traits, Suicide

## Abstract

**Background:** Suicide is a major health problem accounting for 9% of all deaths. Thus, suicide prevention is of particular importance in high-risk groups. Taking care of the individuals who have committed suicide is also considered as a major problem for health professionals. In general, individuals’ personality and attitude toward problems are involved in the incidence of suicide. Human personality is an integrated but complex phenomenon characterized by its extensiveness and large variety of concepts. The present study aimed to evaluate personality traits and coping styles among suicidal and non-suicidal individuals referring to the hospitals and health centers affiliated to Shiraz University of Medical Sciences.

**Methods:** In this cross-sectional study, 100 suicidal patients and 100 non-suicidal individuals (mean age: 27.21 years) were randomly selected and matched in terms of demographic variables. All the participants completed Neuroticism, Extraversion, Openness (NEO) personality questionnaire, Lazarus coping strategies questionnaire, and SCL-90-R. Then, the data were analyzed using T-test, chi-square test, and stepwise liner regression.

**Results:** The study results showed that the highest scores in the case and control groups were related to neuroticism (32.35±3.21) and conscientiousness (36.87±3.26), respectively (P<0.001). The two groups were also compared with respect to the two main types of coping styles. In both coping styles, the mean scores of the control group were higher than those of the case group although the difference was only significant regarding the problem-focused coping style.

**Conclusion:** The results revealed a significant relationship between coping styles and extroversion as well as nervousness. Nervousness and extraversion scores could predict the coping style. In addition, suicidal individuals obtained higher scores in nervousness and emotion-focused coping style compared to the control group.

## Introduction

Studies conducted on the epidemiology of suicide in Iran within the past two decades have shown that the prevalence of suicide has had an increasing trend, especially among the youth.^1^ Psychiatric disorders and psycho-social and biological factors are associated with suicidal attempts.^1^ Other reasons, such as internal and external factors and personality traits, have been stated, as well.

Personality is an abstract intangible concept elicited by a combination of behaviors, thoughts, motivations, emotions, etc. Each person has a unique personality or, better put, a unique set of personality traits resulting in the diversity of human beings. In other words, people are similar with respect to some personality traits and personality can be studied as how people differ from each other or how much they have in common.^2^

Personality traits play an important role in the method with which an individual copes with various problems. The foundations and structures of personality affect an individual’s vulnerability and susceptibility to different stressors. Coping strategies are cognitive and behavioral efforts people make when confronting different stressors. These strategies have a crucial and determining role in people’s mental and physical wellbeing.^3^

Different personalities do not use similar coping strategies in different situations. Effective coping strategies can reduce the intensity of an individual’s reaction to high levels of tension as well as its destructive effects. Stress and tension per se are not as destructive as a negative coping style can be. Therefore, selecting unsuitable strategies upon confronting the stressors can intensify the problem itself, while correct coping mechanisms will yield positive results.^4^ Coping enables people to use their skills and capabilities to manage and overcome difficult situations in life. However, overcoming difficulties does not necessarily mean solving a problem successfully. In other words, coping results from personal evaluation of a specific situation, resources at hand, and being equipped with adaptive coping behavior.^5^

There are two general types of coping styles, namely emotion-focused and problem-focused. Problem-focused coping style consists of any coping behavior that aims to solve a specific problem or eliminate a certain stressor. It is applied when an individual perceives the problem as solvable and the situation as changeable. On the other hand, emotion-focused style is applied when the individual believes that the situation cannot be modified.^6^ These two types comprise various coping strategies. Most of these strategies consist of cognitive processes that reduce the emotional distress and include avoidance, degrading the problem, finding positive points in difficult situations, being different from peers, alcohol consumption, crying, or talking with a friend. These emotion-focused strategies do not often lead to positive psychological or physical results, but the individual will have a better feeling about the situation. Since problem-based coping strategies result in better physical and mental health, they directly reduce the source of stress.^7^

In studies on the personality traits of the individuals who have committed suicide, neuroticism and extraversion were found to influence the suicidal attempts.^8^ Therefore, it is necessary to study and identify different aspects of this bio-psycho-social phenomenon. Studies on personality traits and coping styles of suicidal patients have indicated that many of these individuals had personality disorders.^9^ Most of these people have experienced many psycho-social tensions before committing suicide and tend to use emotion-focused coping strategies when being faced with various problems.^10^ It is necessary to assess variables, such as personality traits and psychological disorders, in such individuals because environmental factor for distress per se cannot define and predict the individuals’ health status.^11^^-^^13^

The present study aims to assess the personality traits and coping strategies in suicidal and non-suicidal individuals in order to determine the common coping strategies and personality traits among the suicidal patients and whether personality traits are related to coping styles in these individuals.

## Materials and Methods

This cross-sectional study was conducted on 100 suicidal patients (case group) between 18 and 30 years who had referred to Faghihi and Ali Asghar Hospitals affiliated to Shiraz University of Medical Sciences, Shiraz, Iran during 2012.

According to the statistical counselor of the study and the study by Shakeri and colleagues, considering power=80% and α=0.05, and using the following formula, a 120-subject sample size was determined for the study (60 subjects in each group). N=[(Z_1_-α/2+Z_1_-β)^ 2 ^(2σ^ 2 ^)]/(μ_1_-μ_2_) in which Z_1_-α/2=1.96, α=0.05 and Z_1_-β=0.84, β=0.20.

Yet, considering the probability of loss during the study, the sample size was increased to 200 subjects (100 in each group).

The patients were matched with 100 normal individuals with respect to sex, age, education level, and marital status. The non-suicidal participants (control group) were selected from 18-30 year-old people accompanying the patients referring to Motahari and Imam Reza clinics, affiliated to Shiraz University of Medical Sciences.

The participants of both groups were selected randomly. In doing so, a number was assigned to each client and then someone was required to select the numbers which were written on pieces of paper.

This study was confirmed by the Ethic Committee of Shiraz University of Medical Sciences. 

The inclusion criteria of the study were being 18-30 years old, not having any debilitating mental illness, history of head trauma, brain tumor, or drug abuse, and being willing to participate in the study. On the other hand, the patients who were in coma because of the toxic effects of the drugs or were not willing to take part in the study were excluded from the research.

The participants’ age, sex, education level, and marital status as well as their emotion- or problem-focused coping style and the relationship between each style and the 5 main personality dimensions were assessed by using the following three questionnaires:

**The Neuroticism, Extraversion, Openness (NEO) short form:** This questionnaire is used for evaluating the five main dimensions of personality in adults. These five personality dimensions are neuroticism, extraversion, openness, agreeableness, and conscientiousness. The questionnaire consists of 60 items rated on a 5-point Likert scale. It also has a section for recording the patients’ demographic characteristics. At the end of the questionnaire, there are 3 questions about the validity of the form that the participants should answer after the test is over. The participants were explained about how to complete the questionnaire and they chose one of the four answers provided for each item. The validity and reliability of this questionnaire were measured by Hagh Shenas reporting an internal consistency of 0.68-0.86. Standardization of this form for the Iranian population was also performed by Haghshenas (2011) and Ashouri (2009) reporting a Cronbach’s alpha of 0.86-0.92.^14^

**Lazarus Coping Strategies Questionnaire: **This questionnaire consists of 66 items beginning with a phrase that motivates the participants to think about the situations. Each item has four choices rated on a 4-point Likert scale ranging from 0-3. The validity and reliability of this questionnaire for the Iranian population was assessed by Feilian (1992), Raafiee (1994), and Tavangar (1994) who respectively reported the correlation coefficients of 0.98, 0.69, and 0.86 using the test-retest method.^15^

**The Symptom Checlist-90-Revised (SCL-90 R): **This checklist is used to screen psychiatric disorders in the patients who have committed suicide in order to determine how many suicidal patients already have signs of psychiatric disorders. This checklist reflects psychological as well as psychiatric signs and is rated on a 5-point Likert scale ranging from 0-4. SCL-90 R evaluates the experienced symptoms within the past 7 days and consists of 9 subscales; i.e., somatization, obsessive-compulsive, interpersonal sensitivity, depression, anxiety, hostility, phobic anxiety, paranoid ideation, and psychoticism. The validity of the questionnaire was revealed to be 97% and its sensitivity, specificity, and reliability were reported as 94%, 98%, and 96%, respectively.^16^

To maintain confidentiality, the patients’ names were not written on top of their forms. 

After obtaining written informed consents from the participants, sufficient information was given to them regarding the study and the checklists. Then, the participants completed the questionnaires in order. To prevent boredom, the questionnaires were handed to the suicidal patients with 2-hour intervals. 

Finally, the data were entered into the SPSS statistical software (version 16) and analyzed using independent t-test, Chi-square test, and stepwise liner regression analysis as appropriated.

## Results

Table 1 shows the frequency of some demographic characteristics. As the table depicts, the patients were matched with respect to their demographic characteristics. In addition, the mean±SD age of the participants in both groups was 27.21±3.15 years. The suicidal patients had committed suicide using toxins and drugs.

**Table 1:** Frequency (%) of some demographic variables in the case and control groups

**Group** **Variable**		**Case** **Frequency (%)**	**Control** **Frequency (%)**
Education level	Below diploma	24 (24)	24 (24)
	Diploma	54 (54)	54 (54)
	Academic degree	22 (22)	22 (22)
Marital status	Single	31 (31)	31 (31)
	Married	65 (65)	65 (65)
	Widowed	1 (1)	1 (1)
	Divorced	3 (3)	3 (3)
Sex	Male	25 (25)	25 (25)
	Female	75 (75)	75 (75)

SCL-90 R checklist was used to for screening the participants with respect to their mental status. In comparison to the control group, the case group participants had obtained higher mean scores in all the nine subscales, especially regarding the depression scores (28.58±14.18 and 19.7±10.66 in the case and control groups, respectively) (P<0.001).

Considering NEO questionnaire, the highest scores in the case and control groups were related to neuroticism (32.35±3.21) and conscientiousness (36.87±3.26), respectively (P<0.001). The two groups were also compared with respect to the two main types of coping styles. In both coping styles, the mean scores of the control group were higher than those of the case group although the difference was only significant regarding the problem-focused coping style (table 2).

**Table 2:** Mean scores of coping style and personality traits in the case and control groups

**Variables**	**Group**	** Mean±SD **	**T-value**	**Sig**
Neuroticism	Case	32.35±3.2	8.75	0.001
	Control	25.29±6.8		
Extraversion	Case	21±6.2	6.58	0.001
	Control	28.66±9.6		
Openness	Case	22.47±6.31	2.09	0.038
	Control	24.24±5.04		
Agreeableness	Case	26.1±3.48	7.86	0.001
	Control	31.47±5.52		
Consciousness	Case	28.5±3.2	8.19	0.001
	Control	36.87±9.5		
Emotion-focused	Case	33.88±13.06	0.852	0.349
	Control	35.44±6.80		
Problem-focused	Case	25.65±9.06	6.598	0.001
	Control	35.43±10.13		

The results revealed a significant correlation between the coping styles and neuroticism as well as conscientiousness. Accordingly, the individuals with higher neuroticism scores used the emotion-focused coping style more frequently.

Additionally, personality traits could be reliable predictors of the individuals’ type of coping styles. In this study, liner stepwise regression analysis was used to assess the prediction rate of personality traits from emotion- and problem-focused coping styles. In the emotion-focused coping style, only neuroticism was entered into the prediction model. Based on the results, neuroticism could significantly predict the emotion-focused coping style (β=0.218; t-value=2.23; P<0.05), and could estimate 0.05% of the variance of emotion-focused coping style. The results have been presented in table 3.

**Table 3:** The results of regression analysis predicting emotion-focused coping styles

	**Sum of Squares**	**Df**	**Mean of Square**	**F**	**Sig**
Regression	313.7	1	313.7	5.00	0.028
Residual	6268.5	100	62.6		
Total	6582.2	101			

In the problem-focused coping style, on the other hand, only openness to experiences was entered into the prediction model and was a significant predictor of problem-focused coping styles (Beta=0.42; t-value=5.05; P<0.001). It could also estimate 0.18% of variance of problem-focused coping style. The results have been shown in table 4.

**Table 4:** The results of regression analysis predicting problem-focused coping styles

	**Sum of Squares**	**Df**	**Mean of Square**	**F**	**Sig**
Regression	2617.18	1	2617.18	25.51	0.001
Residual	12102.7	118	102.56		
Total	14719.96	119			

## Discussion

Personality is an abstract concept. It is similar to a kind of energy in Physics that cannot be observed, but can be distinguished via behavior, thought, motivation, and excitement.^17^^,^^18^

Personality traits exist in everybody providing the basis for behavior conduct. In other words, personality traits establish behaviors. Moreover, personality traits can be used to predict coping styles. 

The findings of the present study revealed a significant difference between the patients who had committed suicide and normal individuals regarding their personality traits, coping styles, and mental health. In other words, suicidal patients have personality traits, such as neuroticism and introversion, use emotion-focused coping styles, and are depressed and physically unhealthy. Our findings are consistent with those of other similar studies comparing suicidal and non-suicidal individuals.^18^

The study findings were also in agreement with those of other studies concerning the psycho-cognitive variables. Human behavior and personality are formed through interaction with the environment and society. Individual differences in human characteristics play a significant role in their behavior and personality. The individuals who commit suicide have neuroticism and emotion focused coping style and they need to be trained regarding the coping styles. Therefore, designing an interventional program for prevention of suicide is highly essential.^19^^-^^21^

In a study on 60 cases who had committed suicide, the researchers found that these people were introverted. Other studies have also confirmed the use of emotion-focused coping style by the individuals committing suicide.^22^

Considering the relationship between the personality traits and coping styles in suicidal individuals, other studies have also indicated that the people who are introverted or obsessive-compulsive tend to use emotion-focused coping styles when they confront stressful situations.^22^

The present study findings showed that the frequency of committing suicide was three folds higher among females compared to males. National and international studies have also confirmed our findings with respect to the role of sex as a risk factor for committing suicide. This might be due to the fact that women experience more stressful situations, are more venerable to such situations, and consider them as uncontrollable.^23^

Nelson and Simmons stated that the youth who had committed suicide used problem-focused coping styles less than their peers. They also concluded that the students suffering from depression used coping styles more frequently. Similarly, another study in Iran revealed that the individuals with mental disorders tended to use emotion-focused coping styles more than problem-focused ones.^22^^,^^24^^,^^25^

In the previous studies, the age range for committing suicide was 15-30 years. When solving a certain problem seems impossible, emotion-focused coping styles are more frequently used because they enable the individuals to tolerate or accept that certain problem and interpret it again in a more tolerable manner.  Using emotion-focused coping styles helps the individuals to regulate the tensions caused by stressful situations as well as independent environmental changes. Based on the learned helplessness theory, depressed people have learned that their reaction and response do not change the result and, consequently, lack cognitive and emotional motivation. After undesirable circumstances, they bring internal justifications that damage their self-esteem. They have a negative image of themselves and the environment, are pessimistic, and use emotion-focused coping styles more often. In order to be treated, they must learn to control their emotions and overcome the environment.^26^^-^^28^

It seems that the view points and attitudes of the youth should be considered as an important factor in preventing suicide. Madlin (2004) stated that in the youth who have committed suicide, attitudes and perceptions are the main factors for selecting inefficient coping styles when facing problems and difficulties. According to Erikson’s theory of psychosocial development, solving difficult situations in the 8 stages of life creates various coping resources for an individual.^29^^-^^31^

By teaching effective coping styles to young people, they will be equipped with skills for managing the stressful situations and overcoming the difficulties.^32^^,^^33^ Yet, coping strategies are not innate skills and must be taught. Considering the close relationship between mental disorders, especially depression and suicide, it is necessary to prevent, diagnose, and treat mental disorders in the youth in order to reduce the rate of suicide among them. Therefore, by determining the NEO scores and predicting the individuals’ coping styles, suicidal attempts can be prevented.

Since human behavior stems from the interaction between the personality traits and the social environment, personality traits have a fundamental role in interpersonal differences and behaviors.^34^^,^^35^ Thus, identifying these traits is necessary for designing the interventional programs targeting the people who have committed suicide. These people might not have learned efficient and adaptive coping styles, because problem-focused coping styles are cognitive-behavioral skills that people learn through training and observing the behavioral models.

One of the limitations of our study was that we only evaluated the people who had committed suicide using drugs and other types of suicidal attempts were not considered. Another limitation was that we only evaluated 18-30 year-old individuals. Thus, different age ranges should also be studied in order to generalize the findings.

## Conclusion

Coping styles can be predicted in suicidal patients using the NEO questionnaire and determining neuroticism. Yet, the difference in neuroticism scores is a more effective predictor of coping styles. Therefore, interventions related to coping styles and effective personality traits along with problem solving training can be effective in preventing suicide. On the other hand, considering the important role of the nursing profession and the fact that the patients spend a long time with nurses during their recovery, they can provide such training for the patients. 

## References

[1] Banakar B (1996). A survey on personality in 11-25 years old suicidal persons. MS.c Thesis.

[2] Sadock BJ, Kaplan HI, Sadock VA, Rezaee F (2007). Aplan&Sadock`s synopsis of psychiatry behavioral sciences /clinical psychiatry.

[3] Vagheei S, Kooshan M (2011). Mental health book.

[4] Bapiri OA, Bahamin GH, Feizolahi A (2010). Survey effect group teaching problem solving on cognitive trait in adolescence suicides. Journal of Ilam Medical University.

[5] Jahangir F, Bazrafshan M, Zangooee AR, Reesi T (2009). Comparison coping style in suicides and non suicides. Hormozgan Medical Journal.

[6] Heidari Pahlavian A, Amirzargar MA, Farhadinasab A, Mahjoub H (2003). Survey and comparison personality in opium addicts with non addicts person. Journal of Hamadan University of Medical Science.

[7] Ashouri A, Habibi Asgarabad M, Torkman Malayeri M, Javan Esmali A (2009). Relationship between suicidal ideation  and personality in substance abuser. Journal of Behavioral Sciences.

[8] Shakeri J, Parvizifard AA, Sadeghi KH, Moradi  R (2005). Personality, stress, coping style and religious attitude in suicides person. Journal of Psychiatry and Clinical Psychology.

[9] Shamsalizadeh N, Afkhamzade A, Mohsen Pour B, Salehian B (2010). Suicide and related factor in kurdestan. Journal of Kurdistan University of Medical Sciences.

[10] Ajilchi B, Naderi A, Ghaemi F (2009). Relationship personality of addicts women with social discipline. Seasonal Journal Social Order.

[11] Moeeni A, Ashkani A, Touhidi M (2002). Survey suicide with drugs and poisonous substance. Medical Research Journal.

[12] Hatami HR, Mohamadi N, Ebrahimi SM, Hatami M (2011). Relationship between locus of control and personality. Tought& Behavior in Clinical of Psychology.

[13] Dafeei M, Dehghani A (2002). Survey and relationship coping style and religious attitude and social activity in infertile pairs. J Shaheed Sadoughi Univ Med Sci.

[14] Haghshenas H (2011). Personality psychology book.

[15] Zeyghami R (1999). A survey stress factor and coping style in students. MS.c Thesis.

[16] Noorbala AA, Arbabi M, Shalbafan AR (2010). Psychological dimentions in patients admitted in Imam Khomeini General Hospital in Tehran. Iran Journal Psychiatry.

[17] Pinnar J, Rothmann S (2007). Occuptional traits and coping styles and suicidal ideation in the South African. Criminal Justice and Behavior Journal.

[18] Must A, Kurgyis E, Szkaliczki A (2012). Personality trait and coping compensate for disadvantageous decision-making in long term alcohol abstinence. Alcohol Alcohol.

[19] Connor Smith JK, Flachsbart C (2007). Relationship between personality and coping: a meta- analysis. J Pers Soc Psychol.

[20] Carrer CS, Smith JC (2010). Personality and coping. Theannual Review of Psychology.

[21] Duhacheck A, Iacobucci D (2005). Consumer personality and coping testing Rival Theories of process. Journal of Consumer Psychology.

[22] Bakhshi Pour A, Mahmood Alilou M, Irani S (2008). The Comparison of personality trait, personality disorders, and problem solving Sterategies in self-introduced Addicts and Normal population. Iranian Journal of Psychiatry.

[23] Farzin Rad B, Asgharnejad Farid A, Yekkeh Yazdandoust R, Habibi Asgarabad M (2010). Comparison of coping sterategies and personality styles in depressed and non-depressed students. Journal of Behavioral Sciences.

[24] Kadivar P, Zahedi F (2007). Coping style with crisis and suicidal attempt in girls students. Social and Welfare Quarterly.

[25] Nelson DL, Simmons BL (2003). Health psychology and work stress A more positive approach. Hand book of occupational health psychology.

[26] Pejušković B, Lečić-Toševski D, Priebe S, Tošković O (2011). Burnout syndrome among physicians - the role of personality dimensions and coping strategies. Psychiatr Danub.

[27] van Eijsden-Besseling MD, Peeters FP, Reijnen JA, de Bie RA (2004). Perfectionism and coping strategies as risk factors for the development of non-specific work-related upper limb disorders (WRULD). Occup Med (Lond).

[28] Wiggins JS (1996). The Five-Factor Model of personality theoretical perspective.

[29] Strutton D, Pelton LE, Lumpkin JR (1995). Personality characteristics and salepeople `s choice of coping startegies. Journal of the Academy of Marketing Science.

[30] Meško M, Karpljuk D, Videmšek M, Podbregar I (2009). Personality profiles and stress coping strategies of military pilots. Psihološka obzorja&Horizons of Psychology.

[31] Berker HV (2009). The relationship between personality and coping styles and stress, anxiety and depression. Msc thesis in psychology.

[32] Chiou PN, Chen YS, Lee YC (2006). Characteristics of Adolescent suicide attempters admitted to an acute psychiatric ward in Taiwan. Chin Med Assoc Journal.

[33] Diaconu G, Turecki G (2009). Family history of suicidal behavior predict impulsive- aggressive behavior level in psychiatric outpatients. Journal of Affective Disorders.

[34] Oquendo MA, Mann JJ (2001). Neuroimaging finding in major depression , suicidal behavior and aggression. Clinical Neuroscience Research Journal.

[35] Rushton JP, Irwing P (2009). A general factor of personality (GFP) from the multidimentional personality questionnaire. Personality and Individual Differences.

